# Unintended consequences of glucagon-like peptide-1 receptor agonists medications in children and adolescents: A call to action

**DOI:** 10.1017/cts.2023.612

**Published:** 2023-08-18

**Authors:** Dan M. Cooper, Mark A. Rothstein, Alpesh Amin, Jan D. Hirsch, Emma Cooper

**Affiliations:** 1 Department of Pediatrics, School of Medicine, Institute for Clinical and Translational Science, University of California at Irvine, Irvine, CA, USA; 2 University of California at Irvine, Institute for Clinical and Translational Science, Irvine, CA, USA; 3 Department of Medicine, School of Medicine, University of California at Irvine, Irvine, CA, USA; 4 School of Pharmacy and Pharmaceutical Sciences, University of California at Irvine, Irvine, CA, USA; 5 Department of Psychiatry, School of Medicine, University of California at Irvine, Irvine, CA, USA

**Keywords:** Pediatric, drug abuse, appetite suppressant, adolescent, energy expenditure, eating disorders, unexpected effects

The purpose of this perspective is to highlight potential unintended and adverse consequences of the increasing use of glucagon-like peptide-1 receptor agonists (GLP-1RAs) in children and adolescents. We propose a set of activities suited in particular to the NIH National Center for Advancing Translational Science (NCATS) network of Clinical and Translational Science Award (CTSA) hubs to mitigate these possible threats to pediatric health. Our apprehension is heightened, ironically, because recent studies have corroborated in children and adolescents the remarkable effectiveness of the GLP-1RAs in the management of type 2 diabetes and, as satiety-enhancing medications, in obesity that had been previously demonstrated in adults. Two studies were published in the impactful *New England Journal of Medicine* [1,2], research motivated, as noted by the authors, in no small measure precisely because the currently approved medications for pediatric obesity and type 2 diabetes have proven to be limited in their effectiveness and fraught with adverse events [3]. Current administration of the GLP-1RAs is predominantly parenteral, but with the progress in the development of oral formulations [4], their increased use in children and adolescents, unsupervised and/or medically supervised, is inevitable. Little attention has been paid to the possible unintended consequences or adverse impact of these medications on children and adolescents during their critical period of growth and development (Table 1).

**Table 1.** Possible unintended consequences of supervised or unsupervised use of oral GLP-1 receptor agonists in children and adolescents

Adverse effect	Reason for concern
Long-term consequences on growth and development	Balance of energy intake and energy expenditure modulate critical growth hormones such as GH and IGF-1. The appetite suppressant, anti-hedonic, and fatigue effects of the GLP-1RA may alter this balance during critical periods of growth and development.
Abuse among patients with diagnosed eating disorders	Pediatric-aged patients (male and female) with eating disorders are known to abuse appetite suppressants.
Abuse among children and adolescents in certain competitive sports (e.g., wrestling, martial arts, gymnastics, ballet)	Children and adolescents involved in school, club, or community sports like wrestling or gymnastics where body weight is a competitive factor are known to engage in unhealthy behaviors to accelerate weight loss.
Unsupervised use for unwarranted self-perception of obesity and overweight, particularly among female adolescents	Inaccurate perception of being overweight in healthy-weight adolescents result in unnecessary weight control behaviors [29]. In children and young adults, supplements sold for weight loss, muscle building, and energy were associated with almost three times the risk for severe medical outcomes compared with vitamins [30].
Excessive or insufficient medical prescription in populations with high prevalence of obesity and poor fitness	Pediatricians have little or no formal training in effective ways to deal with obesity, overweight, or poor physical fitness in their patients. Short-term effectiveness and convenience of drug prescriptions may be well intended but lead to untested effects when used for longer duration during critical periods of growth and development.
Distorted cost-benefit relationship of long-term use in youth	Result in disparities in GLP-1RA availability Promote counterfeit or drug diversion activity. Off-label utilization may not be covered by insurers. Possible lifelong therapy may be cost-prohibitive.

A major element of our concern is that unbalanced and inappropriate reductions in caloric (energy) intake could be induced by GLP-1RA in children and adolescents. Energy in children and adolescents is expended not only on physical activity but also, unlike in adults, on growth and development. The balance of energy intake and energy expenditure influences growth and health across the lifespan. For example, with appropriate levels of exercise and diet during adolescence, bone mineralization is increased and the risk of osteoporosis and pathologic fractures much later in life is lessened [5]. Almost any deviation from healthy levels of physical activity and diet can adversely impact catabolic and anabolic mediators. Seemingly disparate conditions such as adolescents who are normal-weight but physically inactive [6], those who exercise at excessive levels [7,8], and obese children and adolescents [9], all manifest harmful growth patterns and, often, elevated inflammation associated with increased cardiovascular disease risk.

Rapid development of oral-administration formulations of GLP-1RA and the proclivity among adolescents for risk-taking [10] create a perfect storm for potential abuse. Adolescence marks a particularly vulnerable period for the development of self-esteem and satisfaction with one’s own appearance. Lifetime prevalence data in the United States from 2010 indicated that 2.7% of adolescents manifested eating disorders at some point in their lifetime, with females being more than twice as likely as males [11]. Volitional emesis and the use of laxatives or appetite suppressants are not uncommon in youth [12]. In recent years, the rapid expansion of social media has resulted in more youth being exposed to body image ideals and diet culture than ever before. Young users of social media have a higher risk of developing eating disorders [13]. The potential for GLP-1RA abuse was further enhanced by the shutdowns associated with the COVID-19 pandemic that worsened the intractable epidemics of pediatric obesity and poor cardiorespiratory-metabolic fitness, particularly among minority populations of children and adolescents.

Anecdotal clinical experience of our group suggests that there is already widespread knowledge in the pediatric population about the GLP-1RA’s effectiveness as satiety medication aiding weight loss, not helped by apparent widespread use documented in the popular media [14]. Health threats resulting from the explosion of counterfeit drugs are well documented [15] and fueled in part by illegitimate access through the internet [16]. We fear that children and adolescents participating in weight-sensitive activities such as wrestling, bodybuilding, cheerleading, gymnastics, or dance might be particularly vulnerable to unsupervised acquisition and use of these medications.

Adding to our concerns about potential abuse and overuse of the new GLP-1RA medications is the increasing medicalization of pediatric conditions [17], many of which result from environmental and societal rather than biological mechanisms, and the lack of progress in particular made in addressing the environmental and lifestyle issues that have contributed immeasurably to the childhood obesity epidemic. The issue of lifestyles was, in fact, highlighted in particular by Weghuber *et al*. cited above in their publication on semaglutide effectiveness in pediatric obesity [1]. The authors carefully adhered to the “lifestyle interventions” mandated by the FDA and its European counterpart for clinical trials of medications for type 2 diabetes and obesity in the pediatric age range [18,19]. However, the lifestyle intervention guidelines, dating back to 2007–2008, do not address the need to precisely measure dose, frequency, and type of physical activity or diet and do not suggest ways (e.g., cardiopulmonary exercise testing) other than self-report to monitor intervention compliance or fidelity.

We recognize the challenges imposed by implementing truly effective lifestyle interventions in children and adolescents who are obese or suffer from frank type 2 diabetes. By the time such children or adolescents gain access to medical intervention (which is currently limited at best) or are eligible for clinical trials, the clinical trialist and primary care provider must overcome the years of poor diet, sedentary behavior, and lack of available, safe, and supportive venues for vigorous play and exercise that for the majority of these children paved the way for their conditions. In the classic 2002 NEJM study designed to prevent the transition of pre-diabetes to frank type 2 diabetes in adults [20], the authors concluded, “Lifestyle changes and treatment with metformin both reduced the incidence of diabetes in persons at high risk. The lifestyle intervention was more effective than metformin.” We speculate that the very fact that the lifestyle interventions in the recent pediatric study had virtually no effect suggests that their ineffectiveness resulted from insufficient implementation and not lack of biological efficacy.

The pharmacokinetics and pharmacodynamics of certain GLP-1RAs like liraglutide have been studied in both adult and pediatric populations [21]; however, children are not miniature adults and as newer formulations emerge, it cannot be assumed that pharmacokinetics or adverse effects in adults are the same in children or adolescents [22]. We must also consider that pediatric dosage forms may be more expensive than adult formulations [23], there is a potential for lifelong reliance on these medications if started in childhood, and GLP-1RA may require lifestyle interventions [24] to optimize safety, efficacy, and value during growth and development.

The GLP-1RA class of medications will benefit children and adolescents with morbid obesity and type 2 diabetes. We also believe that their overuse and abuse are inevitable. The NCATS emphasis on the science of translation positions the network of CTSA hubs to mitigate these potential threats to child and adolescent health. Attention to real-world data, team science, community engagement, implementation and dissemination, and health disparities will be key in formulating research and policy planning. Accordingly, we propose the following elements as a call to action for CTSA hubs across the nation:

1. Build and support multidisciplinary teams of frontline clinicians, community partners, physiologists, and behavioral and pharmaceutical scientists to address the knowledge gap in GLP-1RA effects in children and adolescents (see Fig. 1).
2. Address the translational bioethics research [25] issues that will result from approval of pediatric formulations of the GLP-1RA medications in particular and, in general, that have evolved from the medicalization of health conditions like pediatric obesity.
3. Engage and improve the quality and accessibility of relevant real-world data such as school-based physical fitness testing (SB-PFT), mandated in sixteen states covering ∼ 60% of American school children [26]. SB-PFT can prove to be an enormously helpful health and fitness metric used to identify regions and communities where poor cardiorespiratory-metabolic fitness and obesity are high, and the possible need for and abuse of GLP-1RA medications are most likely.
4. Work with the FDA and other agencies to update guidelines for lifestyle interventions in pediatric clinical trials that incorporate state-of-the-art approaches to quantifying, monitoring, and evaluating physical activity, adherence to diet, and accurate measurement of body composition beyond the current reliance on the body mass index, a suboptimal metric of overweight and obesity in adolescents [27].
5. Elevate and enhance training of the clinical trial workforce on state-of-the-art understanding of effective lifestyle interventions. Such training should also target primary care pediatricians whose exposure to exercise and nutritional science is currently quite limited [28].
6. Develop, demonstrate, and disseminate learning modules for school personnel (teachers, coaches), parents, school-aged children, primary care pediatricians, and child mental health professionals about the GL1-RA medications, their appropriate uses, and possible abuse.

**Figure 1.**
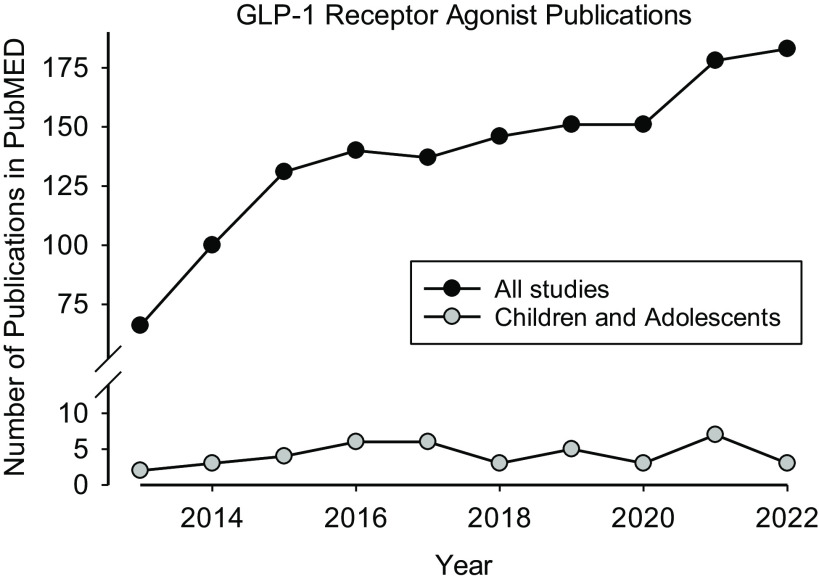
Dearth of research on GLP-1 RAs in children and adolescents. The use of these medications in the pediatric age range is likely to be long term. Little is known about the long-term and lifespan effects of these drugs during critical periods of growth and development.

## References

[1] Weghuber D , Barrett T , Barrientos-Pérez M , et al. Once-weekly semaglutide in adolescents with obesity. New Engl J Med. 2022;387(24):2245–2257. 36322838 10.1056/NEJMoa2208601 PMC9997064

[2] Arslanian SA , Hannon T , Zeitler P , et al. Once-weekly dulaglutide for the treatment of youths with Type 2 Diabetes. N Engl J Med. 2022;387(5):433–443. 35658022 10.1056/NEJMoa2204601

[3] Woodard K , Louque L , Hsia DS. Medications for the treatment of obesity in adolescents. Ther Adv Endocrinol Metab. 2020;11:1–5. 10.1177/2042018820918789 PMC7257846 32523671

[4] Saxena AR , Frias JP , Brown LS , et al. Efficacy and safety of oral small molecule glucagon-like Peptide 1 Receptor agonist danuglipron for glycemic control among patients with Type 2 Diabetes: a randomized clinical trial. JAMA Netw Open. 2023; 6(5):e2314493. 37213102 10.1001/jamanetworkopen.2023.14493 PMC10203889

[5] Nguyen VH. School-based exercise interventions effectively increase bone mineralization in children and adolescents. Osteoporos Sarcopenia. 2018;4(2):39–46. 30775541 10.1016/j.afos.2018.05.002 PMC6362970

[6] Ischander M , Zaldivar Jr. F , Eliakim A , Nussbaum E , Dunton G , Leu SYY , et al. Physical activity, growth, and inflammatory mediators in BMI-matched female adolescents. MedSciSportsExerc. 2007;39(7):1131–1138. 10.1249/mss.0b013e318053e7a2 17596781

[7] Nemet D , Connolly PH , Pontello-Pescatello AM , et al. Negative energy balance plays a major role in the IGF-i response to exercise training. J Appl Physiol. 2004;96(1):276–282. 12949013 10.1152/japplphysiol.00654.2003

[8] Javed A , Tebben PJ , Fischer PR , Lteif AN. Female athlete triad and its components: Toward improved screening and management. Mayo Clin Proc. 2013;88:996–1009. 24001492 10.1016/j.mayocp.2013.07.001

[9] Petek TH , Petek T , Močnik M , Varda NM. Systemic Inflammation, Oxidative Stress and Cardiovascular Health in Children and Adolescents: A Systematic Review. Antioxidants (Basel). 2022;11:894. 35624760 10.3390/antiox11050894 PMC9137597

[10] Winer JM , Yule AM , Hadland SE , Bagley SM. Addressing adolescent substance use with a public health prevention framework: the case for harm reduction. Ann Med. 2022;54:2123–2136. 35900132 10.1080/07853890.2022.2104922 PMC9341337

[11] Merikangas KR , He JP , Burstein M , et al. Lifetime prevalence of mental disorders in U.S. adolescents: results from the national comorbidity survey replication-adolescent supplement (NCS-A). J Am Acad Child Adolesc Psychiatry. 2010;49(10):980–989. 20855043 10.1016/j.jaac.2010.05.017 PMC2946114

[12] Youth Risk Behavior Surveillance — United States, 2011 [Internet]. Available from: https://www.cdc.gov/mmwr/preview/mmwrhtml/ss6104a1.htm#Tab107. Accessed July 19, 2023.

[13] Wilksch SM , O’Shea A , Ho P , Byrne S , Wade TD. The relationship between social media use and disordered eating in young adolescents. Int J Eat Disord. 2020;53(1):96–106. 31797420 10.1002/eat.23198

[14] Maurice JP. Why Ozempic Isn’t Just Getting Celebrities Thin: It’s Killing The Diet Industry Too. New York Post; 2023.

[15] Blumenberg A , Hughes A , Reckers A , Ellison R , Gerona R. Flualprazolam: report of an outbreak of a new psychoactive substance in adolescents. Pediatrics. 2020;146(1):1–5. 10.1542/peds.2019-2953 32581001

[16] Michael White C. Counterfeit drugs: a major issue for vulnerable citizens throughout the world and in the United States. J Am Pharm Assoc. 2021;61(1):e93–e98. 10.1016/j.japh.2020.04.020 32471767

[17] Marty C , Alvey JC , Mann K , Murphy NA. Addressing Over-Medicalization in Children with Medical Complexity. Curr Phys Med Rehabil Rep. 2019;7:6–10.

[18] Food and Drug Administration. Developing products for weight management revision 1 (https://www.fda.gov/regulatory-information/search-fda-guidance-documents/developing-products-weight-management-revision-1). Accessed February 2007.

[19] European Medicines Agency. Clinical evaluation of medicinal products used in weight control — addendum on weight control in children (https://www.ema.europa.eu/en/clinical-evaluation-medicinal-products-used-weight-control-addendum-weight-control-children). Accessed July 24, 2008.

[20] Knowler WC , Barrett-Connor E , Fowler SE , et al. Reduction in the incidence of type 2 diabetes with lifestyle intervention or metformin. N Engl J Med. 2002;346(6):393–403. 11832527 10.1056/NEJMoa012512 PMC1370926

[21] Carlsson Petri KC , Hale PM , Hesse D , Rathor N , Mastrandrea LD. Liraglutide pharmacokinetics and exposure-response in adolescents with obesity. Pediatr Obes. 2021;16(10):e12799. 33963681 10.1111/ijpo.12799 PMC8519033

[22] Verscheijden LFM , Koenderink JB , Johnson TN , de Wildt SN , Russel FGM. Physiologically-based pharmacokinetic models for children: Starting to reach maturation? Pharmacol Ther. 2020;211:107541. 32246949 10.1016/j.pharmthera.2020.107541

[23] Luthra S. Kaiser Health News on PBS. 2017. Why pediatric dosage forms may be more expensive than adult. Available from: https://www.pbs.org/newshour/health/kid-friendly-medications-can-expensive-parents. Accessed July 22, 2023.

[24] Sandsdal RM , Juhl CR , Jensen SBK , Lundgren JR , Janus C , Blond MB , et al. Combination of exercise and GLP-1 receptor agonist treatment reduces severity of metabolic syndrome, abdominal obesity, and inflammation: a randomized controlled trial. Cardiovasc Diabetol. 2023;22(1):41. 36841762 10.1186/s12933-023-01765-z PMC9960425

[25] Rothstein MA. Expanding the role of bioethics in translational science. J Law Med Ethics. 202;50(3):603–607. 36398631 10.1017/jme.2022.99

[26] Krochmal P , Cooper DM , Radom-Aizik S , Lu KD. US school-based physical fitness assessments and data dissemination. J Sch Health. 2021; 91(9):722–729. 34235722 10.1111/josh.13067 PMC9291210

[27] Karchynskaya V , Kopcakova J , Klein D , et al. Is BMI a valid indicator of overweight and obesity for adolescents? Int J Environ Res Public Health. 2020;17(13):4815. 32635439 10.3390/ijerph17134815 PMC7369744

[28] Lu KD , Cooper D , Dubrowski R , Barwick M , Radom-Aizik S. Exploration of barriers and facilitators to implementing best practice in exercise medicine in primary pediatric care-pediatrician perspectives. Pediatr Exerc Sci. 2021;33(4):162–169. 34167088 10.1123/pes.2020-0214

[29] Chung AE , Perrin EM , Skinner AC. Accuracy of child and adolescent weight perceptions and their relationships to dieting and exercise behaviors: a NHANES study. Acad Pediatr. 2013;13(4):371–378. 23830022 10.1016/j.acap.2013.04.011 PMC4130653

[30] Or F , Kim Y , Simms J , Bryn Austin S. Taking stock of dietary supplements’ harmful effects on children, adolescents, and young adults. J Adolesc Health, 2019; 65(4):455–461. doi: 10.1016/j.jadohealth.2019.03.005. 31176525

